# Pan‐Immune‐Inflammation Value Predicts 3‐Month Functional Outcomes in Patients With Acute Ischemic Stroke Treated With Mechanical Thrombectomy

**DOI:** 10.1002/brb3.70397

**Published:** 2025-03-14

**Authors:** Meltem Karacan Gölen, Keziban Uçar Karabulut, Muhammed Kamiloğlu, Aynur Yonar

**Affiliations:** ^1^ Department of Neurology Konya Training and Research Hospital Baskent University Konya Turkey; ^2^ Department of Emergency Medicine Konya Training and Research Hospital Baskent University Konya Turkey; ^3^ Faculty of Science, Department of Statistics Selcuk University Konya Turkey

**Keywords:** acute ischemic stroke, mechanical thrombectomy, pan‐immune‐inflammation value, systemic immune‐inflammation index

## Abstract

**Background:**

The inflammatory response plays a central role in the clinical outcomes of cerebrovascular disease. The aim of this study was to investigate the clinical significance of pan‐immune‐inflammation value (PIV) in patients with acute ischemic stroke after mechanical thrombectomy (MT).

**Methods:**

The study included 201 patients who underwent MT. Blood samples taken from the patients before the procedure were evaluated and inflammation markers were calculated. Severity of stroke was assessed using the National Institute of Health Stroke Scale (NIHSS) scores on admission. Poor 3‐month functional outcome was defined as Modified Rankin Scale (mRS) scores of >2. Ischemic stroke types were classified according to the Trial of Org 10172 in Acute Stroke Treatment (TOAST) classification

**Results:**

In the logistic regression analysis, we observed that PIV was associated with a poor outcome. Post hoc multiple comparison tests revealed statistically significant differences in PIV between the stroke of other determined etiology and small‐vessel occlusion (178.00 vs. 74.89, *p* = 0.015 and *p* < 0.05, respectively), large artery atherosclerosis (178.00 vs. 95.51, *p* = 0.032 and *p* < 0.05, respectively), and cardioembolism (178.00 vs.107.97, *p* = 0.043 and *p *< 0.05) subtypes. There was a moderate positive statistically significant relationship at the 95% confidence level between NIHSS score and PIV (*r* = 0.696, *p* < 0.05).

**Conclusion:**

Our study revealed that PIV predicts a poor 3‐month prognosis in acute ischemic cerebrovascular disease after MT with a significantly better performance than the widely known systemic immune‐inflammation index, systemic inflammation response index, platelet/lymphocyte ratio, and neutrophil/lymphocyte ratio. PIV can be a novel prognostic marker indicating poor prognosis in patients treated with MT.

## Graphical abstract

In our study, high PIV values ​​ were associated with poor outcome.

The positive correlation between PIV and stroke severity.

PIV performs better than SII, SIRI, NLR, and PLR in predicting poor outcomes

As a result, PIV is a superior prognostic marker for stroke patients post‐MT. Potential clinical impact: Helps in early risk stratification and treatment optimization.

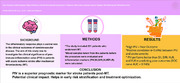

**Abbreviations**
AIS: acute ischemic stroke
CE: cardioembolism
IVT: intravenous thrombolytic therapy
LAA: large artery atherosclerosis
MT: mechanical thrombectomy
NIHSS: National Institute of Health Stroke Scale scores
NLR: neutrophil/lymphocyte ratio
OTT: onset treatment time
PIV: pan‐immune inflammation value
SII: systemic Immune‐Inflammation Index
SIRI: systemic inflammation response index
SOE: stroke of other determined etiology
SUE: stroke of undetermined etiology
SVO: small‐vessel occlusion

## 1 Introduction

Ischemic stroke accounts for 70% of acute cerebrovascular diseases, and approximately 11.6% of deaths worldwide occur due to acute ischemic stroke (AIS), which is among the leading causes of death and disability (Feigin et al. 2021). In the treatment of AIS, intravenous thrombolytic therapy (IVT) has taken an important place among the treatment options recently as it allows the recovery of a possible permanent neurological deficit in eligible patients who reach the hospital within the first 4.5 h after the onset of patient symptoms (Wang et al. 2023). Endovascular mechanical thrombectomy (MT) has been reported to be a good alternative as a treatment option in patients with stroke due to large artery atherosclerosis (LAA), which plays an important role in the etiology of AIS (Wassélius et al. 2022; Li et al. 2022).

The effect of inflammation in acute cerebrovascular disease is particularly important because inflammation plays a prominent role in the pathophysiology of atherosclerosis. Lymphopenia develops due to apoptosis of lymphocytes under the influence of physiologic stress during the inflammatory process, plaque rupture occurs due to increased neutrophil counts, and this whole process leads to atherosclerosis by causing reperfusion injury and plaque remodeling. Increased neutrophil levels stimulate thrombogenesis, leading to inflammation and thrombosis. Activated platelets promote rupture of thrombus formation from atherosclerotic plaques, and activated neutrophils increase the risk of rupture by activating the release of proteolytic enzymes and myeloperoxidase‐like oxidation enzymes. As a result, when ischemic cerebrovascular disease develops, neutrophil, platelet, and monocyte levels increase, and lymphocyte levels decrease in the event of acute stress, and this situation has been found to be associated with the severity of ischemia (Yi et al. 2021).

In this regard, the inflammatory response has been proven to play a role in the development and prognosis of AIS. Recently, systemic immune‐inflammation markers obtained by using neutrophil, lymphocyte, monocyte, and platelet levels in combination as inflammation markers and in predicting prognosis have come to the fore, and studies have revealed that they have stronger predictive value in showing inflammation compared with known parameters such as white blood cells (WBCs), neutrophil/lymphocyte ratio (NLR), and neutrophil and lymphocyte levels as inflammation markers (Yi et al. 2021; Wang et al. 2019).

Increased systemic immune inflammation index (SII), system inflammation response index (SIRI), and PIV indicate increased neutrophil, platelet monocyte ratio, or a decreased lymphocyte‐mediated anti‐inflammatory response. There are limited studies in the literature to determine stroke SII and SIRI levels, but none determine pan‐immune‐inflammation values (PIV).

Previous studies indicated that SII is effective in predicting inflammation and prognosis in many disease groups (Passardi et al. 2016; Hou et al. 2021). A population‐based study examining the relationship between SII and stroke implied that increased SII levels were linked to stroke, but more large‐scale prospective investigations were needed to confirm these findings (Shi et al. 2025). SIRI was defined as a new inflammatory marker by Qi et al. Similarly, SIRI, which is based on a combination of neutrophils, monocytes, and lymphocytes, is a prognostic indicator in patients with malignancy, and it was thought that SIRI and SII could be used to comprehensively evaluate the inflammation status (Qi et al. 2016). In addition to known systemic inflammation markers, PIV has been introduced as a new inflammation marker.

Compared with known immune markers such as SII, SIRI, NLR, and platelet/lymphocyte ratio (PLR), PIV potentially provides a more comprehensive reflection of inflammation. We believe that this is because PIV is calculated by combining counts of the following four main immune cells in the peripheral blood: neutrophils, monocytes, platelets, and lymphocytes. As a new biomarker of immune‐inflammatory response, PIV can better understand patient immune status and improve the prediction of immunotherapy outcomes. Unlike traditional biomarkers, PIV combines multiple inflammatory signals, providing a comprehensive assessment of the immune system and improving prognostic accuracy. PIV, recently introduced as an all‐in‐one cellular immunoinflammatory marker based on blood counts, has prognostic significance in various types of cancer (Baba et al. 2021; Qi et al. 2023).

To our knowledge, the relationship between PIV and prognosis in patients with AIS who underwent MT has not been reported; our study is the first on this subject. We aimed to evaluate the effectiveness of PIV, SII, SIRI, NLR, and PLR in predicting 3‐month prognosis in patients admitted to our stroke center who underwent MT and/or intravenous thrombolysis.

## 2 Materials and Methods

### 2.1 Patients and Data Collection

This study was approved by the Ethics Committee of Baskent University Institutional Review Board. A total of 202 patients admitted to our stroke center with AIS from January 2023 to January 2024 were included in the study. Demographic characteristics, medical history, symptoms, clinical findings, laboratory findings, imaging findings, chronic diseases, previous stroke history, if any, and examination records of the patients were retrospectively evaluated and recorded.

The exclusion criteria were as follows: History of cerebral infarction, and modified Rankin scale (mRS) score of ≥2 points; history of intracranial hemorrhage, aneurysmal subarachnoid hemorrhage, and sinus venous thrombosis; patients with malignant tumors, autoimmune diseases, myeloproliferative and hematologic diseases, severe hepatic and renal insufficiency or unstable vital signs, and current pregnancy; infection or fever 2 weeks before stroke; and chronic inflammation (including rheumatoid arthritis, vasculitis, inflammatory bowel disease). The diagnosis and classification of ischemic stroke were performed by professional neurologists.

AIS diagnoses were made using brain computed tomography (CT), cranial magnetic resonance imaging (MRI), and CT brain angiography. Clinical symptoms were evaluated by neurologists. The Causative Classification of Stroke algorithm was used for the etiologic classification of stroke. Anamnesis of the patients was taken, especially the time of onset of stroke and onset treatment time (OTT) were recorded. Glasgow Coma Scale (GCS) scores were recorded. National Institute of Heath Stroke Scale (NIHSS) data were recorded at the time of admission according to the examination findings and after intravenous thrombolytic and/or MT at the stroke center. Post‐procedure hospitalization period and intracranial hemorrhage causing deterioration in general condition after the procedure and recurrent stroke were recorded. An mRS score between 0 and 2 was considered a good prognosis, and mRS between 3 and 6 was considered a poor prognosis. An unfavorable functional outcome was defined as an mRS of ≥3 at least 3 months after AIS. In addition, 30‐day mortality was evaluated. Ischemic stroke types were classified according to the Trial of Org 10172 in Acute Stroke Treatment (TOAST) classification (Adams et al. 1993).

### 2.2 Stroke Center Intravenous Recombinant Tissue Plasminogen Activator (rt‐PA) Thrombolysis Therapy and Mechanical Thrombectomy

Intravenous thrombolysis was performed at the stroke center, and digital subtraction angiography (DSA) and MT were performed by the interventional radiology unit in appropriate cases according to the time of arrival, clinic, and test results. The standard dose for intravenous thrombolysis was 0.9 mg of the drug per kilogram of body weight. Ten percent of the planned dose was administered as a bolus over 1 min and the remaining 90% as an infusion over 1 h, with a maximum dose of 90 mg. Neurologic examinations and vital signs were monitored during the administration, and in cases of clinical deterioration, brain CT was performed, and patients were followed up for possible hemorrhage.

MT was performed by the interventional radiology unit for occlusion of the internal carotid artery, middle cerebral artery, anterior cerebral artery, or posterior circulation (vertebral artery or basilar artery). For MT procedures, aspiration, retrievable stent, or combined techniques were used depending on the suitability of the case.

### 2.3 Calculation of Systemic Inflammation Markers

Red blood cell (RBC), WBC, neutrophil, lymphocyte, and platelet counts of the same blood sample were obtained within 24 h of admission to calculate the PIV, SII, SIRI, NLR, and PLR values. Laboratory parameters, NLR, PIV, SII, and SIRI indices were compared. WBC, neutrophil, lymphocyte, platelet, and hemoglabin levels were recorded. PIV, SII, SIRI, and NLR were calculated according to the following formulas:

$$\begin{array}{ccc} \mathrm{PIV} & = & \mathrm{neutrophil},x10^{3}/\mathrm{uL}\times \mathrm{platelet},x10^{3}/\mathrm{uL}\\ & & \times \mathrm{monocyte},x10^{3}/\mathrm{uL}/\mathrm{lymphocyte},x10^{3}/\mathrm{uL} \end{array}$$

$$\mathrm{SII}=\mathrm{neutrophil},x10^{3}/\mathrm{uL}\times \mathrm{platelet},x10^{3}/\mathrm{uL}/\mathrm{lymphocyte},x10^{3}/\mathrm{uL}$$

$$\begin{array}{ccc} \mathrm{SIRI} & = & \mathrm{neutrophil},x10^{3}/\mathrm{uL}\times \mathrm{monocyte},x10^{3}/\mathrm{uL}/\mathrm{lymphocyte},\\ & & x10^{3}/\mathrm{uL} \end{array}$$

$$\mathrm{NLR}=\mathrm{neutrophil},x10^{3}/\mathrm{uL}/\mathrm{platelet},x10^{3}/\mathrm{uL}$$

### 2.4 Statistical Analysis

We used the SPSS version 29.0 (SPSS Inc., Chicago, Illinois, USA) and Python software packages for data analysis. The Kolmogorov–Smirnov test was used to check the normality of data distribution. Descriptive statistics for variables with a normal distribution were analyzed using the independent sample *t*‐test and presented as mean ± standard deviation. For variables with a non‐normal distribution, descriptive statistics were analyzed using the Mann–Whitney *U* test or Kruskal–Wallis *H* test and presented as median (interquartile range). Categorical variables were analyzed using the Chi‐square or Fisher's exact test and presented as percentages. To investigate the association between outcome and PIV, SII, SIRI, and NLR, logistic regression analysis was performed. Variance inflation factors (VIFs) were used to examine multicollinearity and significant interactions between independent variables. Independent variables with multicollinearity relations (VIF >5) were eliminated in the logistic regression analysis. Model performance was evaluated using a confusion matrix, a receiver operating characteristic (ROC) curve, and various performance metrics. The relationship between PIV, SII, SIRI, and NLR and NIHSS scores was evaluated using Spearman's correlation test. A *p* value less than 0.05 was considered statistically significant.

## 3 Results

### 3.1 Baseline Characteristics

After excluding certain patients, the study comprised 201 patients, 112 (55.7%) females, and 89 (44.3%) males. The median age of the patients was 74 (IQR: 23–95) years. Upon admission, the median NIHSS score was 8 (IQR: 2–24). The median PIV level was 1033.67 (IQR: 96.47–25146.87), as presented in Table 1.

**TABLE 1:** Demographics and clinical characteristics of the subgroup according to clinical outcomes.

Variable	Total (*n* = 201)	Favorable outcome group (*n* = 89)	Poor outcome group (*n* = 112)	*p*
Age	74 (23–95)	71 (23–92)	75 (41–95)	0.025
Clinical onset minute	120 (30–720)	120 (30–720)	120 (30–720)	0.223
OTT	210 (30–1200)	240 (30–1200)	180 (60–600)	0.149
GCS	12 (3–15)	15 (7–15)	12 (3–15)	<0.001
NIHSS	8 (2–24)	6.72 (2–22)	13.08 (5–24)	0.001*
NIHSS (after treatment)	6 (0–24)	4.63(0–10)	11.26 (2–24)	<0.001
MRS	3 (0–6)	2(0–3)	4 (3–6)	0.000
Hemoglobin	13.40 ± 0.12	13.61 ± 0.17	13.30 ± 0.17	0.219^a^
WBC, 10^9^/L	9.98 (4.60–40.40)	8.94 (4.60–20.10)	10.85 (5.00–40.40)	0.001*
Neutrophil, 10^9^/L	8.20 (2.73–26.00)	5.68 (2.73–15.70)	9.76 (5.29–26.00)	<0.001
Lymphocyte, 10^9^/L	1.29 (0.34–4.62)	1.86 (0.89–4.62)	1 (0.34–2.55)	<0.001
Monocyte, 110^9^/L	0.73 (0.09–2.34)	0.62 ± 0.02	0.87 ± 0.03	<0.001^a^
Platelet count, 10^9^/L	262 (119–560)	215.22 ± 5.29	344.71 ± 8.63	<0.001
PIV	1033.67 (96.47–25146.87)	381.72 (96.47–1756.66)	2647.3 (145.70–25146.87)	<0.001
SII	1575209.33–15093.75)	662.22 (209.33–2195.83)	3293.8 (549.21–15093.75)	<0.001
SIRI	3.90 (0.37–86.61)	1.63 (0.37–5.81)	8.53 (0.92–86.61)	<0.001
NLR	6.12 (0.93–63.68)	3.23 (0.93–7.60)	10.45 (2.22–63.68)	<0.001
Sex				
Female/Male	112(55.7)/89 (44.3)	49 (55.1)/40 (44.9)	63 (56.3)/49 (43.7)	0.887
RtPA				
Yes/No	53 (26.4)/148 (73.6)	23 (43.4)/66 (44.6)	30 (56.6)/82 (55.4)	0.999
Mechanical thrombectomy treatment Yes/No	57 (28.4)/144(71.6)	19 (33.3)/70 (48.6)	38 (66.7)/74 (51.4)	0.059
Hypertension Yes/No	170 (84.6)/31 (15.4)	72 (42.4)/17 (54.8)	98 (57.6)/14 (41.2)	0.239
Diabetes mellitus Yes/No	58 (28.9)/143 (71.1)	33 (56.9)/56 (39.2)	25 (43.1)/87 (60.8)	0.028
Dyslipidemia Yes/No	34 (16.9)/167 (83.1)	12 (35.3)/77 (46.1)	22 (64.7)/90 (53.9)	0.263
Cardiac valve disease Yes/No	9 (4.5)/190 (95.5)	4 (44.4)/84 (44.2)	5 (55.6)/106 (55.8)	0.999
Coronary artery disease Yes/No	79 (39.3)/122 (60.7)	31 (39.2)/58 (47.5)	48 (60.8)/64 (52.5)	0.309
Atrial fibrillation Yes/No	62 (30.8)/139 (69.2)	21 (33.9)/68 (48.9)	41 (66.1)/71 (51.1)	0.065
History of stroke Yes/No	54 (26.9)/147 (73.1)	15 (27.8)/74 (50.3)	39 (72.2)/73 (49.7)	0.003
**Complications**				
No complication	185 (92)	89 (48.1)	96 (51.9)	0.001
Hemorrhage	12 (6)	0 (0)	12 (100)	
Re‐stenosis	4 (2)	0 (0)	4 (100)	
**Toast**				
SA	19 (9.5)	14 (73.7)	5 (26.3)	0.022
LAA	75 (37.3)	36 (48)	39 (52)	
CE	75 (37.3)	26 (34.7)	49 (65.3)	
SOE	2 (1.00)	0 (0.00)	2 (100)	
SUE	30 (14.9)	13 (43.3)	17 (56.7)	

Abbreviations: CE, cardioembolism; GCS, Glasgow Coma Scale; LAA, large artery atherosclerosis; OTT, onset‐to‐treatment time; PIV, pan‐immune‐inflammation value; PLR, platelet‐to‐lymphocyte ratio; SII, systemic immune‐inflammation index; SIRI, systemic inflammation response index; SOE, stroke of other determined etiology; SUE, stroke of undetermined etiology; SVO, small‐vessel occlusion; TOAST, Trial of Org 10172 in Acute Stroke Treatment; WBC, white blood cell count.

### 3.2 Comparison of the Favorable and Poor Outcome Groups

Table 1 summarizes the baseline characteristics of patients in the favorable and poor outcome groups. Out of the initial 201 patients, 112 experienced a poor outcome, and 89 had a favorable outcome. Patients with favorable outcomes were generally younger [71 (23–92) vs. 75 (41–95), *p* < 0.05], and exhibited lower NIHSS (after treatment) [4.63 (0–10) vs. 11.26 (2–24), *p* < 0.001], mRS scores [2 (0–3) vs. 4 (3–6), *p* < 0.001], WBC [8.94 (4.60–20.10) vs. 10.85 (5.00–40.40), *p* = 0.001], neutrophils [5.68 (2.73–15.70) vs. 9.76 (5.29–26.00), *p* < 0.001], monocytes [0.62 ± 0.02 vs. 0.87 ± 0.03, *p* < 0.001], platelets [215.22 ± 5.29 vs. 344.71 ± 8.63, *p* < 0.001], PIV [381.72 (96.47–1756.66) vs. 2647.3 (145.70–25146.87), *p* < 0.001], SII [662.22 (209.33–2195.83) vs. 3293.8 (549.21–15093.75), *p* < 0.001], SIRI [1.63 (0.37–5.81) vs. 8.53 (0.92–86.61), *p* < 0.001], and NLR [3.23(0.93–7.60) vs. 10.45 (2.22–63.68), *p* < 0.001].

Patients in the favorable outcome group demonstrated upper levels of total GCS scores [15 (7–15) vs. 12 (3–15), *p* < 0.001] and lymphocyte [1.86 (0.89–4.62) vs. 1 (0.34–2.55), *p* < 0.001].

Significant differences were also noted in diabetes [33 (56.9) vs. 25(43.1), *p* < 0.001], complications (*p* = 0.001), and TOAST classifications (*p* < 0.001) between the two groups

### 3.3 Association Between Outcome and PIV, SII, SIRI, and NLR

Logistic regression analysis was conducted using the Backward Wald method to examine the relationship between outcome and PIV, SII, SIRI, and NLR. In the 4th model where all parameters were found to be significant, PIV was associated with outcome (OR = 0.995, *p* < 0.001) (Table 2).

**TABLE 2:** Logistic regression analysis results for association between outcome and PIV, SII, SIRI, and NLR.

Model	Parameter	Estimate	Standard error	Odds ratio	*z*	Wald statistic	*p*
1	(Intercept)	7.423	1.338	1673.248	5.547	30.766	<0.001
	PIV	−0.004	0.003	0.996	−1.126	1.267	0.26
	SII	−0.334	0.652	0.716	−0.512	0.262	0.609
	SIRI	−0.069	0.941	0.933	−0.073	0.005	0.942
	NLR	−0.001	0.004	0.999	−0.185	0.034	0.853
2	(Intercept)	7.399	1.296	1634.626	5.708	32.583	<0.001
	PIV	−0.003	0.001	0.997	−2.354	5.541	0.019
	SII	−0.377	0.279	0.686	−1.354	1.832	0.176
	NLR	−0.001	0.001	0.999	−0.981	0.962	0.327
3	(Intercept)	7.278	1.277	1447.646	5.698	32.466	<0.001
	PIV	−0.004	0.001	0.996	−3.749	14.058	<0.001
	SII	−0.309	0.276	0.734	−1.121	1.257	0.262
4	(Intercept)	6.752	1.12	855.816	6.028	36.336	<0.001
	PIV	−0.005	0.001	0.995	−5.521	30.481	<0.001

Abbreviations: PIV, pan‐immune‐inflammation value; PLR, platelet‐to‐lymphocyte ratio; SII, systemic immune‐inflammation index; SIRI, systemic inflammation response index.

**FIGURE 1:**
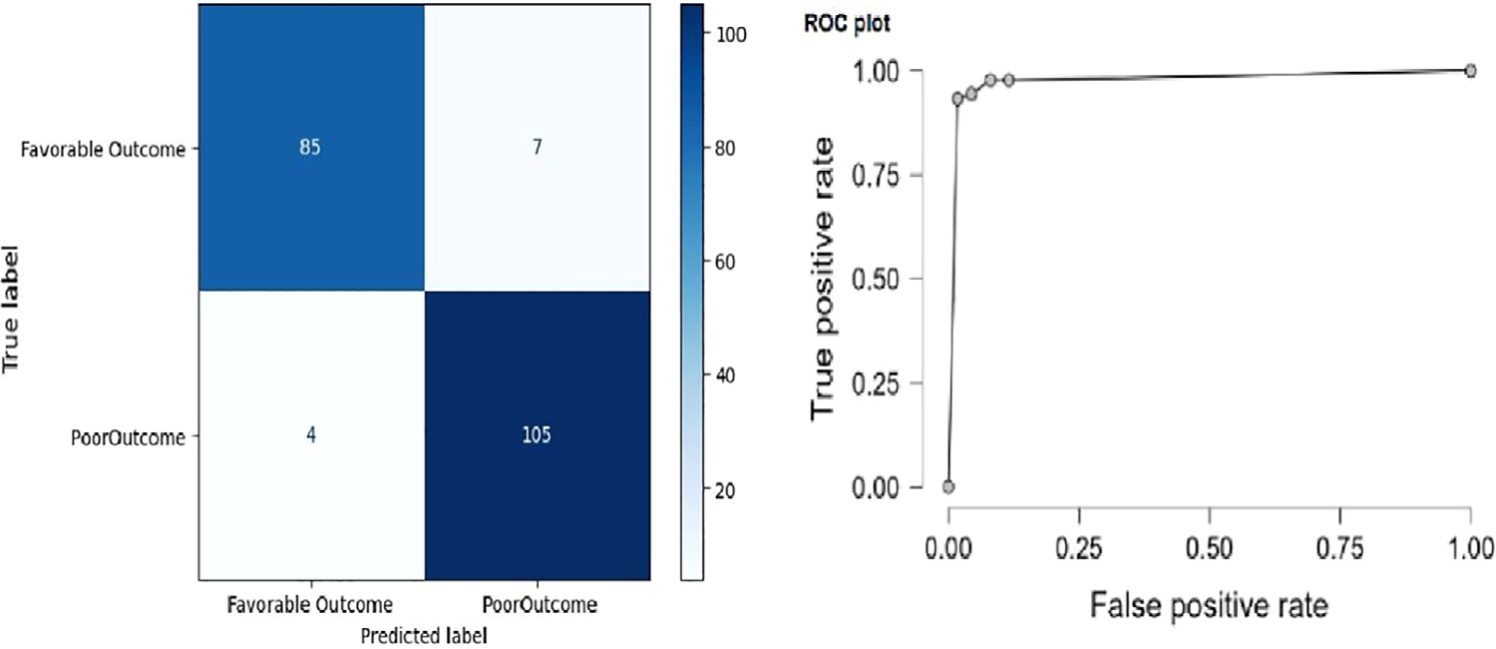
Confusion matrix and ROC curve for Model 4.

Confusion matrix, ROC curve analyses, and performance metrics were used to show the performance of the model in Figure 1 and Table 3. The area under the ROC curve (AUC) is a metric that measures the performance of a classifier model. AUC ranges from 0 to 1, where a value closer to 1 indicates better discrimination ability. In this case, with an AUC of 0.945, the model demonstrates a high ability to discriminate between classes, suggesting strong performance.

**TABLE 3:** Performance metrics for Model 4.

Performance metrics	Accuracy	AUC	Sensitivity	Specificity	Precision	*F*‐measure
**Value**	0.945	0.985	0.955	0.938	0.924	0.939

### 3.3 Comparison of PIV, SII, SIRI, NLR According to TOAST Classification

**FIGURE 2:**
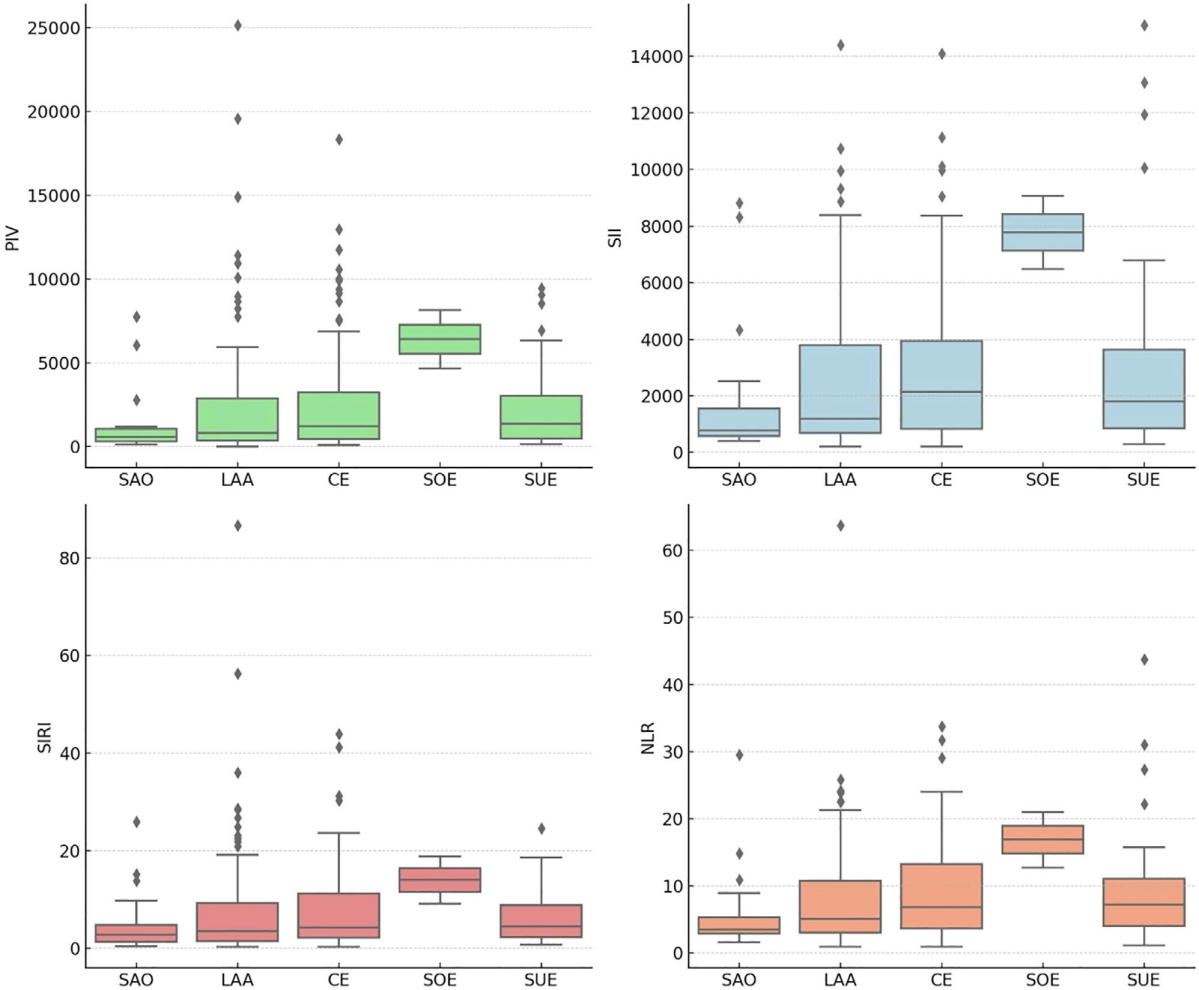
Boxplot graph for PIV, SII, SIRI, and NLR according to TOAST, respectively. CE, cardioembolism; LAA, large artery atherosclerosis; PIV, pan‐immune‐inflammation value; PLR, platelet‐to‐lymphocyte ratio; SII, systemic immune‐inflammation index; SIRI, systemic inflammation response index; SOE, stroke of other determined etiology; SUE, stroke of undetermined etiology; SVO, small‐vessel occlusion; TOAST, Trial of Org 10172 in Acute Stroke Treatment.

The Kruskal–Wallis *H* test results for the comparison of PIV, SII, SIRI, and NLR according to TOAST classification are provided in Table 4. According to the TOAST classification, a difference was found for PIV (*p* < 0.05). Post hoc multiple comparison tests revealed statistically significant differences in PIV between SOE and SVO (178.00 vs. 74.89, *p* = 0.015 and *p* < 0.05, respectively), LAA (178.00 vs. 95.51, *p* = 0.032 and *p* < 0.05, respectively), and CE (178.00 vs. 107.97, *p* = 0.043 and *p* < 0.05) subtypes. Boxplot graphs for PIV, SII, SIRI, and NLR according to TOAST are given in Figure 2, respectively.

**TABLE 4:** PIV, SII, SIRI, and NLR comparison results according to TOAST classification.

Variables TOAST	Mean rank	*p*	
PIV	SVO	74.89	0.048
	LAA	95.51	
	CE	107.97	
	SOE	178.00	
	SUE	108.70	
SII	SVO	77.68	0.142
	LAA	97.47	
	CE	106.03	
	SOE	167.50	
	SUE	107.57	
SIRI	SVO	79.37	0.192
	LAA	97.35	
	CE	105.87	
	SOE	163.00	
	SUE	107.50	
NLR	SVO	74.47	0.052
	LAA	94.96	
	CE	108.99	
	SOE	169.00	
	SUE	108.40	

Abbreviations: CE, cardioembolism; LAA, large artery atherosclerosis; PIV, pan‐immune‐inflammation value; PLR, platelet‐to‐lymphocyte ratio; SII, systemic immune‐inflammation index; SIRI, systemic inflammation response index; SOE, stroke of other determined etiology; SUE, stroke of undetermined etiology; SVO, small vessel occlusion; TOAST, Trial of Org 10172 in Acute Stroke Treatment.

### 3.4 Association Between NIHSS and PIV, SII, SIRI, and NLR

Spearman's rho correlation analysis was conducted to examine the association between NIHSS and PIV, SII, SIRI, and NLR. There was a moderate positive statistically significant relationship at the 95% confidence level between NIHSS score and PIV (*r* = 0.696, *p *< 0.05), SII (*r* = 0.696, *p *< 0.05), SIRI (*r* = 0.684, *p *< 0.05), and NLR (*r* = 0.717, *p *< 0.05) (Table 5).

**TABLE 5:** Spearman's rho correlation results.

	PIV	SII	SIRI	NLR
NIHSS	0.696^**^	0.723^**^	0.684^**^	0.717^**^

Abbreviations: NIHSS, National Institute of Heath Stroke Scale; PIV, pan‐immune‐inflammation value; PLR, platelet to lymphocyte ratio; SII, systemic immune‐inflammation index; SIRI, systemic inflammation response index. ** p < 0.01

## 4 Discussion

Our study evaluated the association of systemic immune‐inflammation markers with poor 3‐month outcomes in patients with acute cerebrovascular disease who underwent MT. PIV values were higher in the poor 3‐month outcome group than in the favorable outcome group, and we found that high PIV values had a statistically significant relationship in predicting poor 3‐month outcomes in patients who underwent MT. In our study, we applied logistic regression analysis using the Backward Wald method to evaluate the association between inflammatory biomarkers such as PIV, SII, SIRI, NLR, and PLR and poor 3‐month outcomes. As a result, we detected a statistically significant association with PIV in Model 4. Moreover, the evaluation of Model 4 used a confusion matrix and ROC curve (AUC 0.945), indicating that PIV exhibited high performance in predicting poor outcomes. Our study is the first to evaluate the prediction of poor 3‐month outcomes using PIV in patients with acute cerebrovascular disease undergoing MT.

When we look at pathophysiology to understand the role of systemic inflammatory markers in cerebrovascular events, the results obtained suggest that when tissue damage occurs after acute ischemic cerebrovascular disease, molecules released by necrotic cells are thought to activate immune cells in the central nervous system. Neutrophils are the first to be released as an immune response when brain ischemia develops. Infiltrating neutrophils can release a number of pro‐inflammatory mediators, such as matrix metalloproteinases (MMPs), thus aggravating brain inflammation. Neutrophils are associated with stroke severity and function outcome in patients with AIS, as evidenced by previous studies (Anrather and Iadecola 2016; Iadecola and Anrather 2011).

Increased neutrophil counts and released proinflammatory cytokines cause plaque rupture, reperfusion injury, and plaque remodeling, leading to atherosclerosis, which is critical in the development of carotid artery stenosis. It is known that when ischemia develops, lymphopenia develops due to increased numbers of neutrophils and apoptosis of lymphocytes due to acute stress, and studies evaluating this condition have reported that it is related to the severity of ischemia (Yi et al. 2021). Increased neutrophil levels increase platelet levels and cause aggregation, additionally, neutrophils also stimulate thrombogenesis by affecting tissue factors. Thus, neutrophil increases and correlated increases in platelet levels lead to inflammation and thrombosis. Activated platelets release the rupture of thrombus formation from the atherosclerotic plaque. Activated neutrophils increase the risk of rupture by activating the release of proteolytic enzymes and myeloperoxidase‐like oxidation enzymes. Eventually, ruptured plaque formation leads to an ischemic stroke. Histopathologic examination of ruptured plaques causing ischemic stroke or stenosis shows increased neutrophil levels, which supports this finding (Nasr et al. 2009). In AIS, damaged brain tissue often exhibits neutrophil infiltration, and patients may experience increased circulating neutrophil counts. These phenomena correlate with stroke severity, infarct size, and patient prognosis (McDonald et al. 2012).

As is known, PIV contains more parameters than NLR, PLR, SII, and SIRI: neutrophils, platelets, monocytes, and lymphocytes. Both the adhesion of platelets and secretion of procoagulant substances by platelets play important roles in the development and progression of atherosclerosis. Platelets, neutrophils, and monocytes are also very important in atherosclerosis. In particular, neutrophils contribute to the formation of all atherosclerotic plaque processes both directly by invading the plaque and indirectly through the proteolytic enzymes and arachidonic acid they secrete. Considering the contributions of peripheral blood cells to coronary microanatomy, there is a strong biological rationale for the division of monocytes, neutrophils, and platelets into lymphocytes in the PIV score. In a recent study, the predictive efficacy of preoperative PIV was analyzed and it was reported to be superior to NLR, PLR, and SII in predicting in‐hospital and long‐term mortality in patients with ST‐elevation myocardial infarction (STEMI) (Kaplangoray et al. 2024).

PIV is a marker of inflammation that has recently aroused the curiosity of researchers and has been evaluated in studies to investigate its efficacy. In a study investigating patients who received IVT after acute ischemic attacks, the relationship between PIV and 3‐month outcomes was evaluated, and it was reported that high PIV values were independently associated with poor 3‐month outcomes. It was shown in the same study that PIV, similar to other systemic inflammation markers such as PLR, NLR, and SII, could predict adverse outcomes after IV thrombolysis (Wang et al. 2023). In recent years, the focus of research on PIV has focused mainly on its applications for prognosis and therapeutic outcomes in oncologic patients (Corti et al. 2021; Provenzano et al. 2023) Fuca et al. evaluated the efficacy of PIV in patients with metastatic colorectal cancer (mCRC) and they identified PIV as a novel immune inflammatory biomarker in patients with mCRC and demonstrated that PIV was a stronger predictor of survival than SII and PLR in patients undergoing first‐line treatment for mCRC (Fucà et al. 2020). The results of these studies support our study, and we believe that PIV will serve as a marker of inflammation and help in predicting prognosis.

In support of the findings of our study, Han et al. found a statistically significant strong correlation between high PIV values and delayed ischemic stroke in patients with delayed ischemic stroke after intracranial hemorrhage secondary to aneurysm (Han et al. 2024). Similarly, supporting our findings that PIV has a better performance in predicting poor outcomes at 3 months compared with other inflammatory markers, Liu et al. also emphasized that PIV predicted prognosis in STEMI with a better performance than SII (Liu et al. 2023).

In our study, we observed that high SII, SIRI, NRL, and PLR indices were statistically significantly higher in the group with a poor outcome at 3 months with a poorer performance compared with PIV. Hou et al. demonstrated a close link between SII and the severity of AIS, suggesting that SII might be more suitable and effective than other inflammation markers such as NLR and PLR in stroke assessment (Hou et al. 2021).

Weng et al. found that patients with AIS tended to have higher SII values compared with the healthy controls. Higher SII was correlated with a severe stroke. Multivariate logistic regression analysis demonstrated that SII was an independent predictor of poor outcomes at 3 months (Weng et al. 2021).

Another study evaluating clinical outcomes in patients with AIS undergoing MT using SIRI showed that the group with good clinical outcomes who underwent MT for AIS had a lower mean SIRI (2.3) than the group with poor clinical outcomes (3.8), and patients with lower SIRI (<2.9) had better mRS scores and less symptomatic intracranial hemorrhage. In multivariate regression analysis, a SIRI of <2.9 was an independent prognostic predictor associated with favorable clinical outcome (OR: 2.27, 95% CI: 1.29–5.17, P 1–4: 0.019) (Yi et al. 2021)

TOAST is the classification of etiology in patients with acute ischemic cerebrovascular disease, and when the relationship between TOAST classification and ischemic stroke etiologic subtypes and inflammation markers was evaluated, we observed a statistically significant difference between PIV and subtypes (*p* < 0.05). Post hoc multiple comparison tests revealed statistically significant differences in PIV between SOE and SVO (178.00 vs. 74.89, p = 0.015 and p<0.05, respectively), LAA (178.00 vs. 95.51, *p* = 0.032 and *p* < 0.05, respectively), and CE (178.00 vs. 107.97, *p* = 0.043 and *p* < 0.05, respectively) TOAST subtypes. We found no similar studies evaluating the relationship between PIV and stroke subtypes in the literature. To clarify this issue, we believe that future studies investigating whether there is a difference between TOAST classification subtypes and PIV according to etiology will be illuminating in this field, bringing a different perspective to the literature.

In a study comparing inflammatory markers in patients who received IVT in acute ischemia with healthy individuals, it was reported that age, current smoking, AF, previous stroke, initial NIHSS scores, and high SII were significantly associated with poor outcomes at 3 months, as seen in additional variable regression analyses. In our study, in the group of patients who received MT and had poor outcomes, age, pretreatment, and post‐treatment NIHSS scores were observed to be high, apart from inflammatory markers. In our study, no difference was observed between the groups in terms of sex (Weng et al. 2021).

NIHSS scores are calculated based on the patient's neurologic examination findings, and an increase in this score may be a sign of deepening examination findings, an increased likelihood of permanent sequelae, and poor prognosis. We evaluated the relationship between PIV, SII, SIRI, NLR, PLR, and NIHSS scores, which we found to be effective in predicting a poor 3‐month outcome. Evaluating the relationship between NIHSS and PIV, SII, SIRI, and NLR using Spearman's rho correlation analysis, we observed a moderate positive statistically significant relationship between NIHSS score and PIV (*r* = 0.696, *p* < 0.05), SII (*r* = 0.696, *p* < 0.05), SIRI (*r* = 0.684, *p* < 0.05), and NLR (*r* = 0.717, *p* < 0.05) at the 95% confidence level. Considering the objective findings of clinical deterioration according to the level of NIHSS, it can be predicted that there may be improvement with mild–moderate–severe disability, and may even result in mortality. This positive correlation between PIV and high NIHSS, which is based on objective examination findings and evaluation, strongly suggests that PIV is reliable for predicting poor prognosis and will guide physicians.

In a study by Yang et al., the median NIHSS score was 15 (range, 12–18) and the median SII was 820.9 × 109/L (range: 473.1–1345.2). The most likely reason for these results is that Yang et al.’s cohort enrolled more patients with severe stroke who required endovascular treatment, which also lends support to the perspective that elevated SII is strongly associated with stroke severity in patients with AIS (Yang et al. 2022).

## 5 Limitations

This study has some potential limitations. First, this is a single‐center retrospective study and the results are limited by the sample size and study population. The study did not include frequently known markers such as interleukins and plasma factors. We evaluated PIV, SII, SIRI, NLR, and PLR as inflammatory markers according to blood parameters taken at admission and did not analyze the fluctuation behavior of markers with repeated dynamic measurements. We think that the dynamic evaluation of parameters with repeated measurements during hospitalization in future studies will contribute to the literature.

## 6 Conclusions

Our study revealed that PIV predicts a poor 3‐month prognosis in acute ischemic cerebrovascular disease after MT with a significantly better performance than the widely known SII, SIRI, PLR, and NLR. Furthermore, its positive correlation with NIHHS scores supported its ability to predict clinical deterioration. We think that these markers, which can predict prognosis in the AIS MT patient group with noninvasive, cost‐effective, and easily accessible laboratory parameters, are guiding and will be helpful for physicians in a targeted patient follow‐up treatment plan.

## Author Contributions

**Meltem Karacan Gölen**: resources; project administration, software, formal analysis, data curation, supervision, methodology, validation, visualization, writing – review and editing, writing – original draft, funding acquisition, investigation, conceptualization. **Keziban Uçar Karabulut**: data curation, supervision, methodology, conceptualization, validation, investigation, funding acquisition. **Muhammed Kamiloğlu**: conceptualization, investigation, data curation, methodology, funding acquisition. **Aynur Yonar**: software, formal analysis, project administration, data curation.

## Ethics Statement

All procedures followed were in accordance with the ethical standards of the committee responsible for human experimentation (institutional and national) and with the Helsinki Declaration of 1975, as revised in 2008. Ethics committee approval has been granted from our institution. This study was approved by the Ethics Committee of Baskent University Medical and Health Sciences Research Council.

## Consent

The principal author has received consent forms from the participants in this study and has them on file.

## Conflicts of Interest

The authors declare no conflicts of interest.

### Peer Review

The peer review history for this article is available at https://publons.com/publon/10.1002/brb3.70397.

## Data Availability

The data that support the findings of this study are available from the corresponding author upon reasonable request.
